# Detection rate of fluorine-18 prostate-specific membrane antigen-1007 PET/CT for prostate cancer in primary staging and biochemical recurrence with different serum PSA levels: A systematic review and meta-analysis

**DOI:** 10.3389/fonc.2022.911146

**Published:** 2022-07-22

**Authors:** Xue Liu, Tao Jiang, CaiLiang Gao, HuiTing Liu, Yu Sun, Qiao Zou, Rui Tang, WenBing Zeng

**Affiliations:** ^1^ PET-CT Center, Chongqing University Three Gorges Hospital, Chongqing, China; ^2^ Department of Nuclear Medicine, The First People’s Hospital of Huaihua City, Hunan, China

**Keywords:** prostate cancer, biochemical recurrence, meta-analysis, ^18^F-PSMA-1007, PET/CT, prostate-specific antigen

## Abstract

**Background:**

We performed a systematic review and meta-analysis to evaluate the detection rate (DR) of fluoro-prostate-specific membrane antigen (^18^F-PSMA-1007) PET/CT in patients with different serum prostate-specific antigen (PSA) levels in the setting of primary staging of prostate cancer (PCa) or biochemically recurring PCa.

**Methods:**

A comprehensive electronic literature search of the PubMed, Embase, and Cochrane Library databases was conducted in accordance with the PRISMA statement. This study was registered in the PROSPERO database (registration number: CRD42022331595). We calculated the DR of ^18^F-PSMA-1007 PET/CT in PCa.

**Results:**

The final analysis included 15 studies that described 1,022 patients and 2,034 lesions with ^18^F-PSMA-1007 PET/CT in PCa. The DR of ^18^F-PSMA-1007 PET/CT in patients with PCa in primary staging ranged from 90% to 100%, with a pooled estimate of 94% (95% CI: 92%–96%). The DR of ^18^F-PSMA-1007 PET/CT in patients with PCa in BCR ranged from 47% to 100%, with a pooled estimate of 86% (95% CI: 76%–95%). The DRs of PSA levels >2.0, 1.1–2.0, 0.51–1.0, and ≤0.5 ng/ml detected by ^18^F-PSMA-1007 PET/CT in a patient-based analysis were 97% (95% CI: 93%–99%), 95% (95% CI: 88%–99%), 79% (95% CI: 68%–88%), and 68% (95% CI: 58%–78%), respectively.

**Conclusion:**

This meta-analysis concluded that ^18^F-PSMA-1007 PET/CT had a high application value for prostate cancer, including primary tumors and biochemical recurrence. The DR of ^18^F-PSMA-1007 PET/CT was slightly higher in primary prostate tumors than in biochemical recurrence.

**Systematic Review Registration:**

https://www.crd.york.ac.uk/prospero/, identifier CRD42022331595.

## 1 Introduction

Prostate cancer (PCa) is the second most common malignancy in men, excluding non-melanoma skin cancers such as basal and squamous cell carcinomas (1, 2). Typically, PCa patients do not exhibit characteristic clinical symptoms during the early stages of the disease; therefore, by the time PCa is diagnosed, many patients are already advanced in the disease and the tumor cannot be removed (3). Therefore, early diagnosis and treatment are important for PCa. Between 27% and 53% of all patients undergoing radical prostatectomy or radiation therapy develop a rising prostate-specific antigen (PSA) level (PSA recurrence) (4). Importantly, patients with PSA recurrence after radical prostatectomy or primary radiation therapy have different risks of subsequent PCa-specific mortality (4). A recent study investigated the impact of biochemical recurrence (BCR) on hard endpoints and concluded that patients experiencing BCR are at an increased risk of developing distant metastases and PCa-specific and overall mortality (5).

The precise staging of PCa by imaging methods is essential for proper disease management, as treatment options differ for localized PCa, locally advanced PCa, or metastatic disease (6). Prostate-specific membrane antigen (PSMA) is a transmembrane glycoprotein with glutamate carboxypeptidase activity (7). Prostate-specific membrane antigen expression is highly upregulated in advanced, metastatic, and poorly differentiated prostate cancers and increases with tumor aggressiveness; on the other hand, the overexpression of PSMA has not been found in benign prostatic diseases (8). Fluorine-18-PSMA-1007 (^18^F-PSMA-1007) positron emission tomography/computed tomography (PET/CT) is an advanced imaging modality used to assess PCa (9). PET/CT images of the salivary glands, liver, gallbladder, prostate, kidney, and small intestine have a physiological uptake of ^18^F-PSMA-1007; a positive result can also be found in areas with localized abnormal radioactivity uptake, such as in lymph nodes and bones, which can be an indication of metastases (10). Compared with ^68^Gallium-PSMA-11 (^68^Ga-PSMA-11), the most used PSMA imaging agent, ^18^F-PSMA-1007 has many advantages (6, 11, 12). First, ^18^F is produced by a cyclotron, which ensures that ^18^F-PSMA-1007 can be synthesized stably and in large quantities. However, the utility of [^68^Ga] Ga circumvents the need for an on-site cyclotron since it is produced from a ^68^Ge/^68^Ga generator (13). Second, the ^18^F-PSMA-1007 has a longer half-life (110 min) than the ^68^Ga-PSMA-11 (68 min), which facilitates distribution to other regions (11). Third, the deficiency of ^68^GA-PSMA-11 is that it is excreted mainly through the urinary system. If the tracer accumulates in the urinary tract, it may affect the diagnosis of local recurrence after radiotherapy (12). However, ^18^F-PSMA-1007 mainly focuses on hepatobiliary excretion, and the low urine activity can avoid this effect, which is conducive to the display of recurrence and metastasis. Finally, the low positron energy, long half-life, and rapid clearance *in vivo* of ^18^F-PSMA-1007 are convenient for a delayed scan. It can obtain higher tumor-to-background images and is more sensitive in the detection of recurrence than ^68^Ga-PSMA-11 (14, 15).

According to several publications (15–17), ^18^F-PSMA-1007 PET/CT tests are highly valuable for detecting prostate cancer primary lesions and biochemical recurrences. One study (4) involving an intraindividual comparison of prostate cancer patients with ^18^F-PSMA-1007 and ^18^F-fluorodeoxyglucose found that the former had a higher detection rate for primary lesions than the latter [100% (21/21) vs. 67% (14/21)]. For extra-prostatic lesions, the former showed a true positive rate of 60% and the latter 79%. Based on the ^18^F-PSMA-1007 PET/CT results of Giesel et al. (16), 204 (71.3%) of PCa patients showed evidence of recurrence. The percentages of PSA levels greater than or equal to 2, 1 to less than 2, 0.5 to less than 1, and 0.2 to less than 0.5 ng/ml detected by PET/CT were 94.0%, 90.9%, 74.5%, and 61.5%, respectively. Using ^18^F-PSMA-1007 PET/CT, German researchers (15) analyzed 100 cases of pathologically confirmed biochemically recurrent prostate cancer. Among patients with ≤0.5, 0.51–1.0, 1.1–2.0, and >2.0 ng/ml PSA levels, the pathological scanning rates were 86%, 89%, 100%, and 100%, respectively. As a result of the small sample sizes, regional differences, and differing PSA levels, the results of these studies were highly heterogeneous. For this reason, to evaluate the value of ^18^F-PSMA-1007 PET/CT in prostate cancer, it is important to carry out a meta-analysis or systematic review of the previous studies. Despite several published meta-analyses (18–20) assessing the rate of detecting BCR using ^18^F-PSMA-1007 PET/CT, no studies evaluated the efficacy of ^18^F-PSMA-1007 PET/CT for both primary staging and biochemical recurrence in PCa patients with different serum PSA levels.

Therefore, the aim of this meta-analysis and systematic review was to evaluate the application value of ^18^F-PSMA-1007 PET/CT in patients with different serum PSA levels in the setting of primary staging of PCa or biochemically recurring PCa.

## 2 Materials and methods

This meta-analysis was in accordance with the Preferred Reporting Items for Systematic Reviews and Meta-Analyses (PRISMA) statement (see **Supplementary Material** for the PRISMA 2020 Checklist). This study was registered in the PROSPERO database (registration number: CRD42022331595).

### 2.1 Data sources and search strategy

We performed electronic literature searches of the PubMed, Embase, and Cochrane Library databases for English-language articles from the earliest available date of indexing through 30 September 2021. We also manually searched the reference lists of the identified publications to identify additional studies. The following keywords were used for the selection of studies: PSMA, prostate-specific membrane antigen, prostate cancer, prostate recurrence, positron imaging, PET, and ^18^F-PSMA-1007.

### 2.2 Study selection

The inclusion criteria for the relevant studies were as follows: a) ^18^F-PSMA-1007 PET/CT was used to identify and characterize PCa; b) subjects were diagnosed with PCa by histopathology, imaging examinations, or clinical follow-up; c) sufficient data to calculate detection rate (DR) of ^18^F-PSMA-1007 PET/CT in PCa were reported; and d) analyses were performed on a per-patient or per-lesion basis.

The exclusion criteria were as follows: a) overlapping papers; b) review articles, animal experiments, editorials or letters, comments, and conference proceedings; c) a lack of access to the full text; d) insufficient data to assess detection rate from individual studies; and e) a sample size of fewer than 10 patients or lesions.

### 2.3 Data extraction

In this study, the lesion-based analyses included local recurrence, lymph node, and bone and soft tissue lesions. In patient-based studies, the presence of lesions can be used as a covariate analysis. During data extraction, a positive ^18^F-PSMA-1007 scan was defined as follows: intraprostatic lesions were defined as positive if the tracer uptake was focal and higher than the surrounding prostate tissue (15, 21). Other soft tissue and bone metastases were judged as positive when there were obvious morphological changes; meanwhile, the corresponding lesions showed increased radiotracer uptake above normal surroundings (4, 22).

A data abstraction sheet was developed. Two researchers (XL and TJ) independently assessed the collected data that included basic information (authors, publication year, and country), study design (prospective or retrospective), patient characteristics, imaging purpose (initial stage or BCR), sample size (patients or lesions), imaging agent (^68^Ga-PSMA-11 or ^18^F-PSMA-1007), administered activity, level of PSA, and Gleason score for characterizing PCa. In cases of disagreement, a consensus was reached on inclusion or exclusion by discussion, and if necessary, a third researcher (CG) was consulted.

### 2.4 Quality assessment

The methodological quality of the included studies was critically appraised based on the modified Quality Assessment of Diagnostic Accuracy Studies version 2 (QUADAS-2) (23), as recommended by the Cochrane Collaboration. Each item was evaluated as “high,” “low,” or “unclear.” Each paper was scored independently by two evaluators (XL and TJ), and any discrepancies were resolved. The Review Manager software (The Cochrane Collaboration, version 5.3.5, London, United Kingdom) was used to assess the quality.

### 2.5 Statistical analysis and data synthesis

In this study, the data of every eligible study were collected. Descriptive statistics (such as mean, standard deviation, and count) were used to summarize continuous variables, while percentage and count were used for categorical variables. The primary objective was to estimate the DR with a 95% confidence interval (95% CI). Detection rate was defined as the ratio between the number of patients or lesions with at least one suspected lesion detected by imaging facility and the total number of PCa patients who underwent the scan. A bivariate normal random-effects model for measures was used to analyze and pool the diagnostic performance of previous studies (24). Heterogeneity was analyzed using the *χ* ^2^ test, with a *P*-value of less than 0.05 suggesting heterogeneity. In addition, the *I* ^2^ statistic was adopted to evaluate the degree of heterogeneity (25). Based on Cochrane’s handbook, a rough classification of the *I* ^2^ index is as follows: low (0%–40%), moderate (30%–60%), substantial (50%–90%), and considerable variability (75%–100%). The value of *P <*0.05 or *I* ^2^ >50% indicated that there was greater heterogeneity in the specimens (26). Based on the results, the random-effects model was used for further analysis; otherwise, a fixed-effect model was performed. Meanwhile, when there was substantial statistical heterogeneity, we performed subgroup analysis to identify potential sources of bias (27). As described by Deeks and colleagues (28), we examined the possibility of publication bias by using an effective sample size funnel plot and a regression test of asymmetry. Tests for significance were two-tailed, with a statistically significant *P*-value threshold of 0.05. All statistical analyses were carried out using Stata version 16.0 software (StataCorp LP, College Station, TX, USA).

## 3 Results

### 3.1 Literature search and study selection

**Figure 1:**
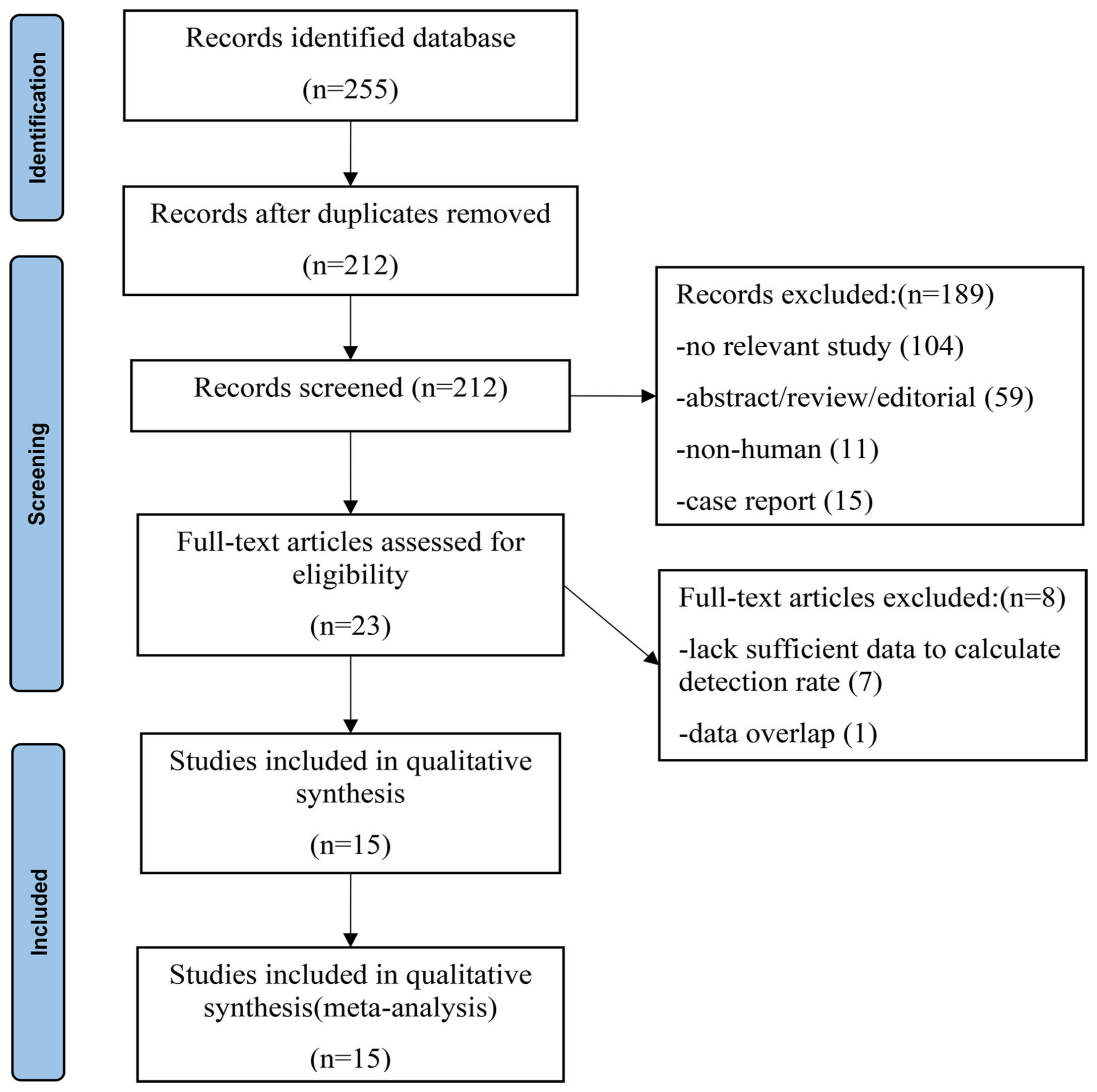
Flowchart of the search for eligible studies on ^18^F-PSMA-1007 PET/CT in patients of prostate cancer.

After a comprehensive computerized search was performed and the reference lists were extensively cross-checked, our study identified 255 records. After reviewing titles and abstracts, 128 records were excluded because they were non-human studies, duplicated reports, reviews, editorials, conference abstracts, or small case series. Additionally, 104 unrelated abstracts were removed. By reading the full texts, seven articles were eliminated because of a lack of sufficient information to calculate the detection rate. Two literature studies (22, 29) were published by the same institution, and the data were duplication. Therefore, only data from the latest article (22) were extracted for meta-analysis. Finally, 15 studies met the inclusion and exclusion criteria, all of which were subjected to a systematic review and meta-analysis. No other articles were found after screening the references of these articles. The detailed process of literature screening is shown in **Figure 1**.

### 3.2 Characteristics of the included studies

The major characteristics of the 15 studies (4, 11, 15–17, 21, 22, 30–37) included in the meta-analysis are described in **Tables 1**, **2**. The 15 articles were published between 2017 and 2021, consisting of 12 retrospective studies (75%) and three prospective studies (25%) (21, 22, 33).

**Table 1:** Basic study and patient characteristics.

Author	Year	Country	Study design	No. of patients/lesions	Age (years)	Imaging purpose	Type of patients evaluated	Median (range)PSA values at PET/CT (ng/ml)	Gleason score
Zhou et al. (4)	2021	China	R	21/124	Median:66	Initial stage	Patients with BCRPCa previously treated with ADT (81%) or RP (52%)	41.20 (5.00–200.00)	≤6: 0%, 7: 42%, ≥8: 58%
Rauscher et al. (11)	2020	Germany	R	102/371	Mean: 71 ± 8	Biochemical recurrence	BCRPCa	0.87 (0.20–13.59)	6–7: 61.8%, 8–10: 38.2%
Rahbar et al. (15)	2018	Germany	R	100/NR	Mean: 68.75 ± 7.6	Biochemical recurrence	Patients with BCRPCa previously treated with RP (92%) or RT (45%) or ADT (27%)	1.34 (0.04–41.3)	≤6: 8%, 7: 56%, ≥8: 36%
Kesch et al. (17)	2017	Germany	R	10/372	Median: 67 (62–77)	Initial stage	Patients with PPCa	13.1 (5.8–40.0)	≤6: 0%, 7: 30%, ≥8: 70%
Trägårdh et al. (31)	2021	Sweden	R	39/118	Mean: 65 ± 5.6	Initial stage	Patients with PPCa	NR	NR
Kuten et al. (21)	2019	Israel	P	16/145	Median: 68.5	Initial stage	Patients with PPCa	6.35 (5.1–10.9)	≤6: 0%, 7: 81%, ≥8: 19%
Malaspina et al. (22)	2021	Finland	P	79/218	Median: 72	Initial stage	Patients with PPCa	Median 12 (3–2,000)	≤6: 100%, 7: 0%, ≥8: 0%
Privé et al. (30)	2020	Netherlands	R	53/46	NR	Initial stage	Patients with PPCa	12 (7.7–20)	≤6: 9%, 7: 36%, ≥8: 55%
Wondergem et al. (32)^a^	2021	Netherlands	R	69/NR	NR	Initial stage	Patients with PPCa	14.7 (2.4–577)	≤6: 0%, 7: 94%, ≥8: 0%, unknown: 6%
						Biochemical recurrence	Patients with BCRPCa previously treated with RP (33.3%) or RT (66.7%)	2.4 (0.4–7.8)	NR
Giesel et al. (16)	2019	Germany	R	251/NR	Median: 70 (48–86)	Biochemical recurrence	Patients with BCRPCa previously treated with RT after RP (43.8%) or ADT (53.4%%)	10.9 (0.6–250)	≤6: 5.2%, 7: 49.8%, ≥8: 33.1%, unknown: 11.2%
Witkowska-Patena et al. (33)	2019	Poland	P	40/NR	Mean: 69 ± 7	Biochemical recurrence	Patients with BCRPCa previously treated with RP (80%) or RT (20%)	0.7 (0.01–2.0)	Mean 7.1 ± 1, median 7 (5–9)
Sachpekidis et al. (34)	2019	Germany	R	17/NR	Median: 66	Biochemical recurrence	Patients with BCRPCa previously treated with RP or RT (100%)	1.2 (0.2–237.3)	≤6: 4%, 7: 44%, ≥8: 24%, unknown: 28%
Dietlein et al. (35)	2020	Germany	R	27/NR	Mean: 67.2 ± 7.8	Biochemical recurrence	Patients with BCRPCa previously treated with RP (93%) or RT (7%)	0.3–27.7	NR
Ahmadi Bidakhvid et al. (36)	2021	Belgium	R	175/580	Mean: 69 ± 8.8	Biochemical recurrence	Patients with BCRPCa previously treated with RP (78%) or RT (35.9%) or high-intensity focused ultrasound (0.7%) or ADT (93.3%)	Median 1.6 (0.07–429)	≤6: 8%, 7: 49%, ≥8: 43%
Morawitz et al. (37)	2021	Germany	R	23/60	Mean: 71 ± 8.5	Biochemical recurrence	BCR after RP (100%)	1.5 (0.2–7.0)	NR

a This study evaluated both the primary staging of prostate cancer and the biochemical recurrence.

P, prospective; R, retrospective; NR, not reported; PPCa, primary prostate cancer; BCR, biochemical recurrence; ADT, androgen-deprivation therapy; RP, radical prostatectomy; RT, radiation therapy.

**Table 2:** Technical aspects of ^18^F-PSMA-1007 in the included studies.

Author	Modality	Radiotracer	Radiotracer injection activity^a^ (mean)	Time interval between radiotracer injection and image acquisition (mean)	Modality manufacturer	Scanning scope	Other imaging performed for comparison
Zhou et al. (4)	PET/CT	^18^F-PSMA-1007	348 ± 52 MBq	180 min	Biograph mCT-64 PET/CT scanner (Siemens)	From the vertex to the mid-thigh	^18^F-FDG PET/CT
Rauscher et al. (11)	PET/CT	^18^F-PSMA-1007	325 ± 40 MBq	94 ± 22 min	Biograph mCT scanner (Siemens Medical Solutions)	NR	^68^Ga-PSMA-11 PET/CT
Rahbar et al. (15)	PET-CT	^18^F-PSMA-1007	338.02 ± 33.31 MBq	Median 120 min	Siemens mCT Scanner (Siemens Healthcare, Knoxville, TN, USA)	From the lower limbs to the skull	–
Kesch et al. (17)	PET/CT	^18^F-PSMA-1007	NR	60 min, delay 180 min	Biograph mCT Flow Scanner (Siemens)	NR	mpMRI
Trägårdh et al. (31)	PET/CT	^18^F-PSMA-1007	4.0 ± 0.4 MBq/kg	120 ± 6 min	Discovery MI (GE Healthcare, Milwaukee, WI, USA)	From the skull base to the mid-thigh	–
Kuten et al. (21)	PET/CT	^18^F-PSMA-1007	4 MBq/kg	60 min	Discovery 690 PET/CT system (GE Healthcare)	From the tip of the skull to the mid-thigh	^68^Ga-PSMA-11 PET/CT
Malaspina et al. (22)	PET/CT	^18^F-PSMA-1007	250 MBq	60 min	Discovery MI digital PET/CT system (GE Healthcare, Milwaukee, WI, USA)	From the vertex to the mid-thigh	MRI
Privé et al. (30)	PET/CT	^18^F-PSMA-1007	250 MBq	90 ± 10 min	Biograph mCT 4-ring, 40-slice TOF PET/CT Scanner (Siemens)	NR	MRI
Wondergem et al. (32)	PET/CT	^18^F-PSMA-1007	324 MBq	90 min	Biograph‐16 TruePoint PET/CT (Siemens Healthcare, Knoxville, USA)	From the skull base to the inguinal region	^18^F-DCFPyL PET/CT
Giesel et al. (16)	PET-CT	^18^F-PSMA-1007	301 ± 6.46 MBq	92 ± 26 min	Biograph mCT Flow Scanner (Siemens Medical Solutions)	NR	–
Witkowska-Patena et al. (33)	PET-CT	^18^F-PSMA-1007	296 ± 14 MBq	95 ± 12 min	Dedicated hybrid PET/CT system (Discovery 710; GE Healthcare, Chicago, IL, USA)	From the top of the head to the mid-thigh	^18^F-FCH PET/CT
Sachpekidis et al. (34)	PET-CT	^18^F-PSMA-1007	Median 237 MBq	60 min	Dedicated PET/CT system (Biograph mCT, 128S, Siemens, Erlangen, Germany)	From the skull to the feet	–
Dietlein et al. (35)	PET/CT	^18^F-PSMA-1007	159 ± 31 MBq	NR	NR	NR	–
Ahmadi Bidakhvid et al. (36)	PET/CT	^18^F-PSMA-1007	3 MBq/kg	81 ± 16 min	Discovery MI-4 PET/CT (GE)	From the vertex to the upper thigh	–
Morawitz et al. (37)	PET/CT	^18^F-PSMA-1007	229 ± 27 MBq	NR	Biograph mCT 128 (Siemens Healthineers, Erlangen, Germany)	From the skull base to the mid-thigh	^68^Ga-PSMA-11 PET/CT

NR, not reported; mpMRI, multiparameter magnetic resonance imaging; ^68^Ga-PSMA, Gallium-68; ^18^F, fluorine-18; PET/CT, positron emission tomography/computed tomography; ^18^F-FCH, fluorine-18-fluorocholine; PSMA, prostate-specific membrane antigen; FDG, fluorodeoxyglucose; DCFPyL, 2-(3-{1-carboxy-5-[(6-[(18)F]fluoro-pyridine-3-carbonyl)-amino]-pentyl}-ureido)-pentanedioic acid.

a Activity (mean activity of the radiotracer applied in MBq; NR, not recorded; reported target dose in MBq/kg).

Seven studies (4, 17, 21, 22, 30–32) assessed the primary staging of prostate cancer. Nine studies (11, 15, 16, 32–37) assessed the biochemical recurrence of prostate cancer. One study (32) evaluated both the primary staging of prostate cancer and the biochemical recurrence.

All studies used PET/CT as an imaging modality. Three studies (17, 22, 30) simultaneously evaluated ^18^F-PSMA-1007 PET/CT and magnetic resonance imaging (MRI). The imaging agents ^18^F-PSMA-1007 and ^68^Ga-PSMA-11 were compared simultaneously in three studies (11, 21, 37). Nearly half of the studies were from Germany (46.7%), and the other studies were from the Netherlands (30, 32), Israel (21), Belgium, Finland (29), Sweden (31), and China (4), respectively.

In total, there were 1,022 PCa patients and 2,034 PCa lesions in the included studies, and the ages of the patients ranged from 48 to 86 years. The number of cases in each study ranged from 10 to 251. The serum PSA levels ranged from 0.01 to 2,000 ng/ml. We conducted all analyses based on per-patient and/or per-lesion data. Unfortunately, only three (15, 16, 33) eligible studies have evaluated the serum PSA grouping.

### 3.3 Risk of bias and applicability

**Figure 2:**
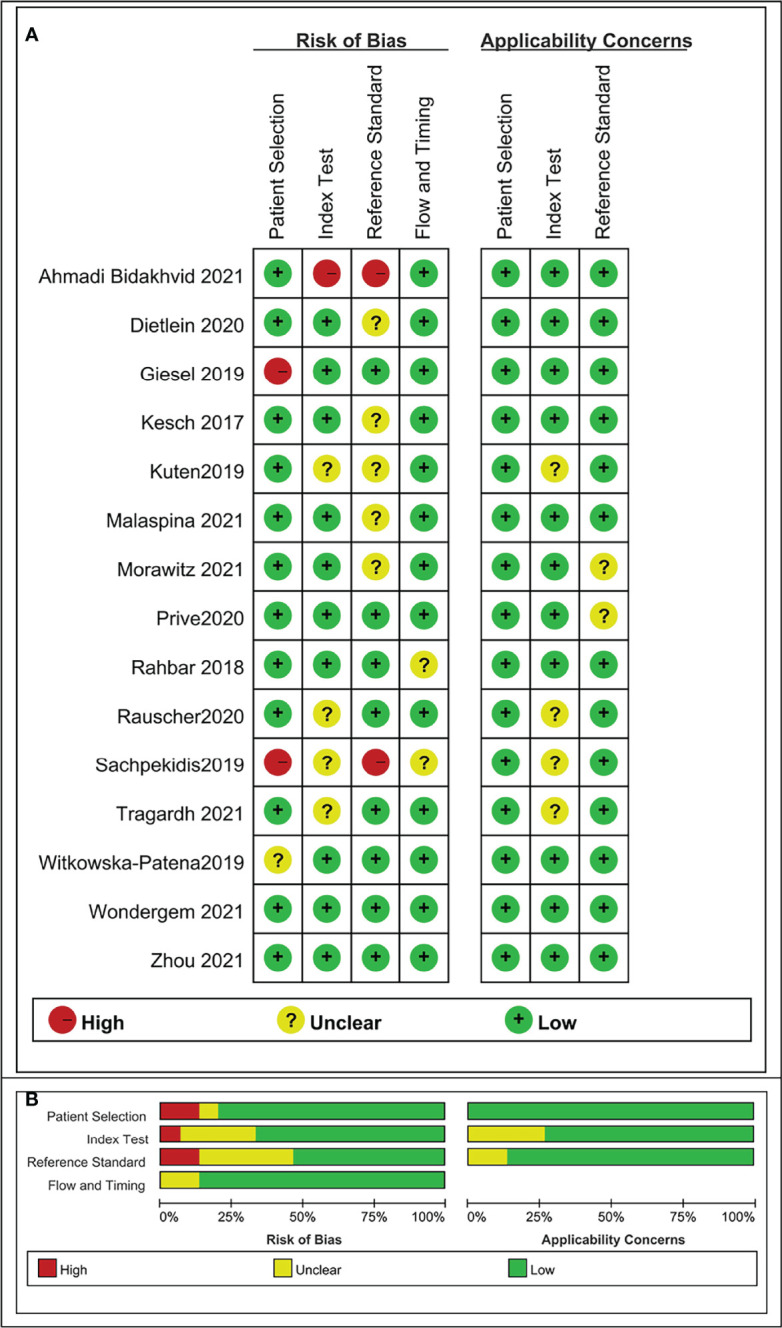
Risk of bias and applicability concerns the summary **(A)** and graph **(B)** of the studies included in the systematic review according to the QUADAS-2 tool.

The risk of bias and applicability concerns for the included studies were assessed using QUADAS-2, as shown in **Figure 2** and **Supplementary Table 1**. All the included studies were of moderate to high quality.

### 3.4 Quantitative analysis (meta-analysis)

#### 3.4.1 18F-PSMA-1007 PET/CT for prostate cancer in primary staging

**Figure 3:**
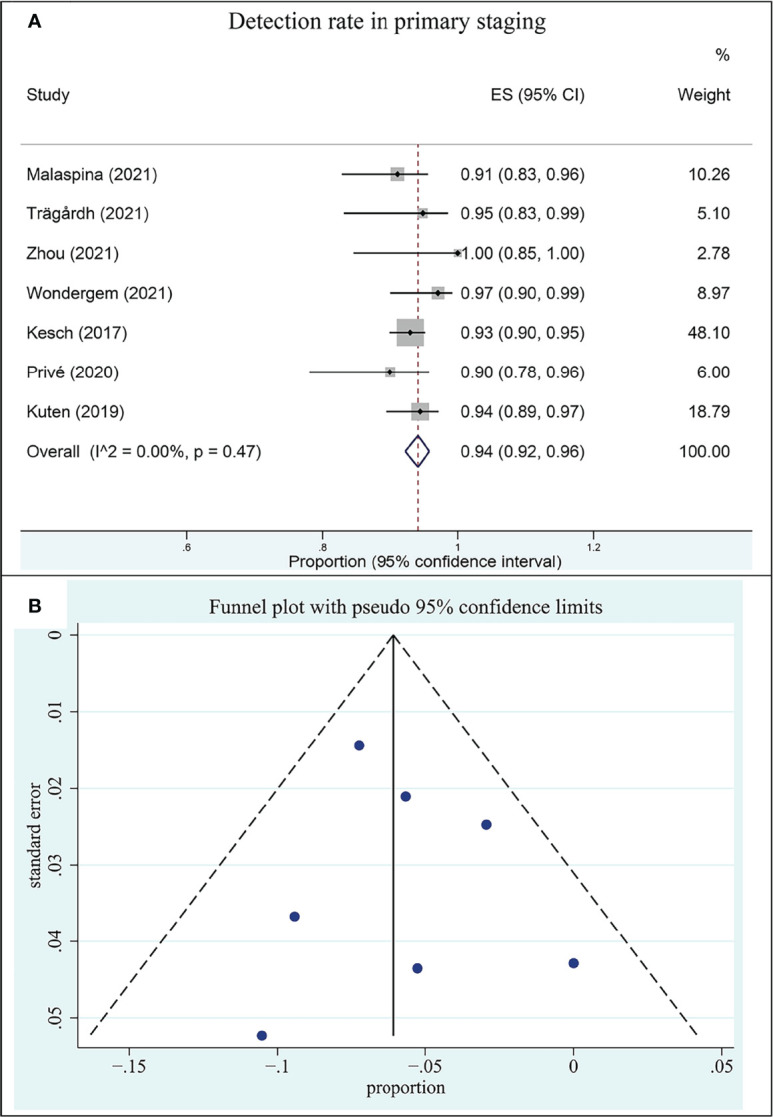
Plot of the pooled detection rate of ^18^F-PSMA-1007 PET/CT for prostate cancer in primary staging **(A)** and related funnel plot for publication bias assessment (**B**).

Seven included studies assessed the ^18^F-PSMA-1007 PET/CT in the setting of primary staging. The DR of ^18^F-PSMA-1007 PET/CT in patients with PCa ranged from 90% to 100%, with a pooled estimate of 94% (95% CI: 92%–96%) (**Figure 3A**). There was no heterogeneity between studies (*I* ^2^: 0.00%).

The funnel plot for publication bias assessment is shown in **Figure 3B**. Egger’s regression intercept for DR pooling was 0.16 (95% CI: −0.36 to 0.69, *P* = 0.460), also indicating that publication bias was absent.

##### 3.4.1.1 Per patient-based or per lesion-based analysis

Four studies (4, 22, 31, 32) assessed the DR of ^18^F-PSMA-1007 PET/CT in a patient-based analysis, with a range of 91% to 100% and a combined estimate of 96% (95% CI: 91%–99%) (**Supplementary Figure 1**). There was no heterogeneity between studies (*I* ^2^: 22.13%).

Six studies (4, 17, 21, 22, 30, 31) assessed the DR of ^18^F-PSMA-1007 PET/CT in a lesion-based analysis, with a range of 53% to 94% and a combined estimate of 81% (95% CI: 66%–92%) (**Supplementary Figure 1**). The included studies were statistically heterogeneous in their estimate of DR (*I* ^2^: 96.47%).

The DR of ^18^F-PSMA-1007 PET/CT for PCa in primary staging was significantly different between patient-based and lesion-based analysis (*P* = 0.02).

##### 3.4.1.2 18F-PSMA-1007 PET/CT vs. MRI

Three studies (17, 22, 30) simultaneously compared the DR of ^18^F-PSMA-1007 PET/CT with MRI for PCa in primary staging in a lesion-based analysis. The pooled DR of ^18^F-PSMA-1007 PET/CT vs. MRI was 88% (95% CI: 79%–95%) vs. 81% (95% CI: 65%–94%), respectively (**Supplementary Figure 2**). There was no significant difference between the two groups (*P* = 0.409).

#### 3.4.2 ^18^F-PSMA-1007 PET/CT for prostate cancer in BCR

**Figure 4:**
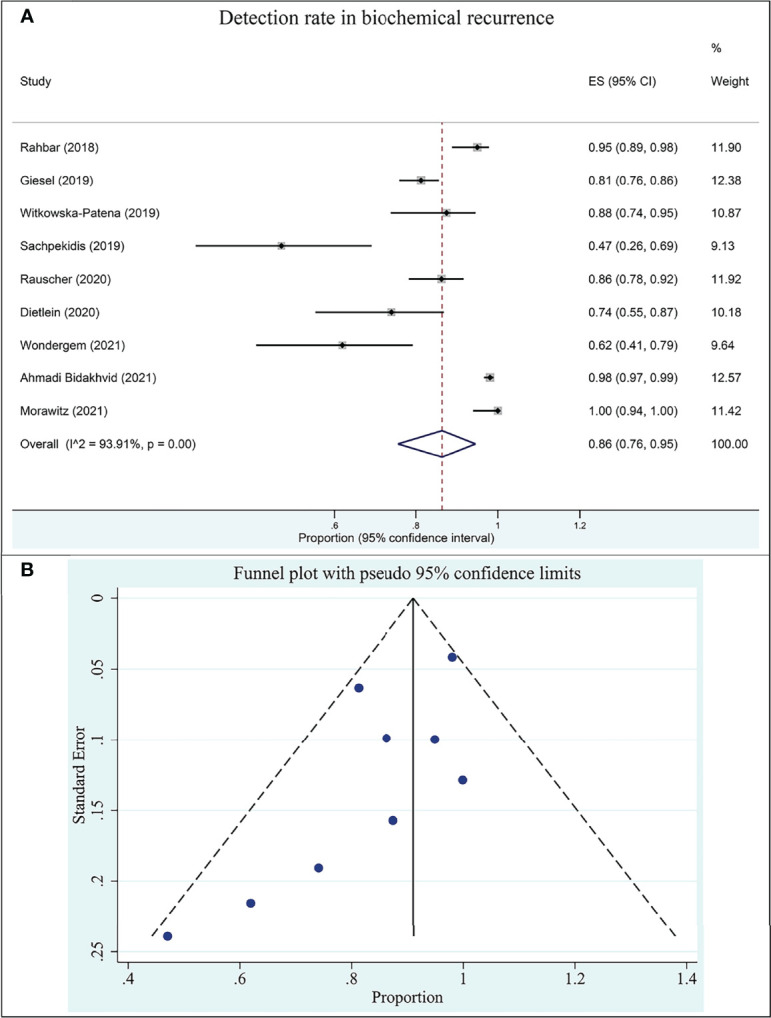
Plot of the pooled detection rate of ^18^F-PSMA-1007 PET/CT for prostate cancer in biochemical recurrence **(A)** and related funnel plot for publication bias assessment (**B**).

Nine studies assessed the DR of ^18^F-PSMA-1007 PET/CT for prostate cancer in BCR in this group. The DR of ^18^F-PSMA-1007 PET/CT in patients with PCa ranged from 47% to 100%, with a pooled estimate of 86% (95% CI: 76%–95%) (**Figure 4A**). The included studies were statistically heterogeneous in their estimate of DR (*I* ^2^: 93.91%).

The funnel plot for publication bias assessment is shown in **Figure 4B**. Egger’s regression intercept for DR pooling was −2.70 (95% CI: −5.81 to 0.41, *P* = 0.079), also indicating that publication bias was absent.

##### 3.4.2.1 Without serum PSA grouping based on patient or lesion analysis

Nine studies (11, 15, 16, 32–37) assessed the DR of ^18^F-PSMA-1007 PET/CT for prostate cancer in BCR based on patient analysis without serum PSA grouping, with a range of 47% to 95% and a pooled estimate of 82% (95% CI: 74% to 88%) (**Supplementary Figure 3**). The included studies were statistically heterogeneous in their estimate of DR (*I* ^2^: 76.92%).

Four studies (11, 16, 36, 37) assessed the DR of ^18^F-PSMA-1007 PET/CT in a lesion-based without serum PSA grouping, with a range of 33% to 100% and a combined estimate of 78% (95% CI: 33%–100%) (**Supplementary Figure 3**). The included studies were statistically heterogeneous in their estimate of DR (*I* ^2^: 99.61%).

There was no significant difference in the DR of ^18^F-PSMA-1007 PET/CT for PCa in BCR between patient-based and lesion-based analyses (*P* = 0.863).

##### 3.4.2.2 Serum PSA subgroup based on patient analysis

Due to limited information, the pooled analysis was performed only for patient-based studies in the subgroup analysis performed with serum PSA.

**Figure 5:**
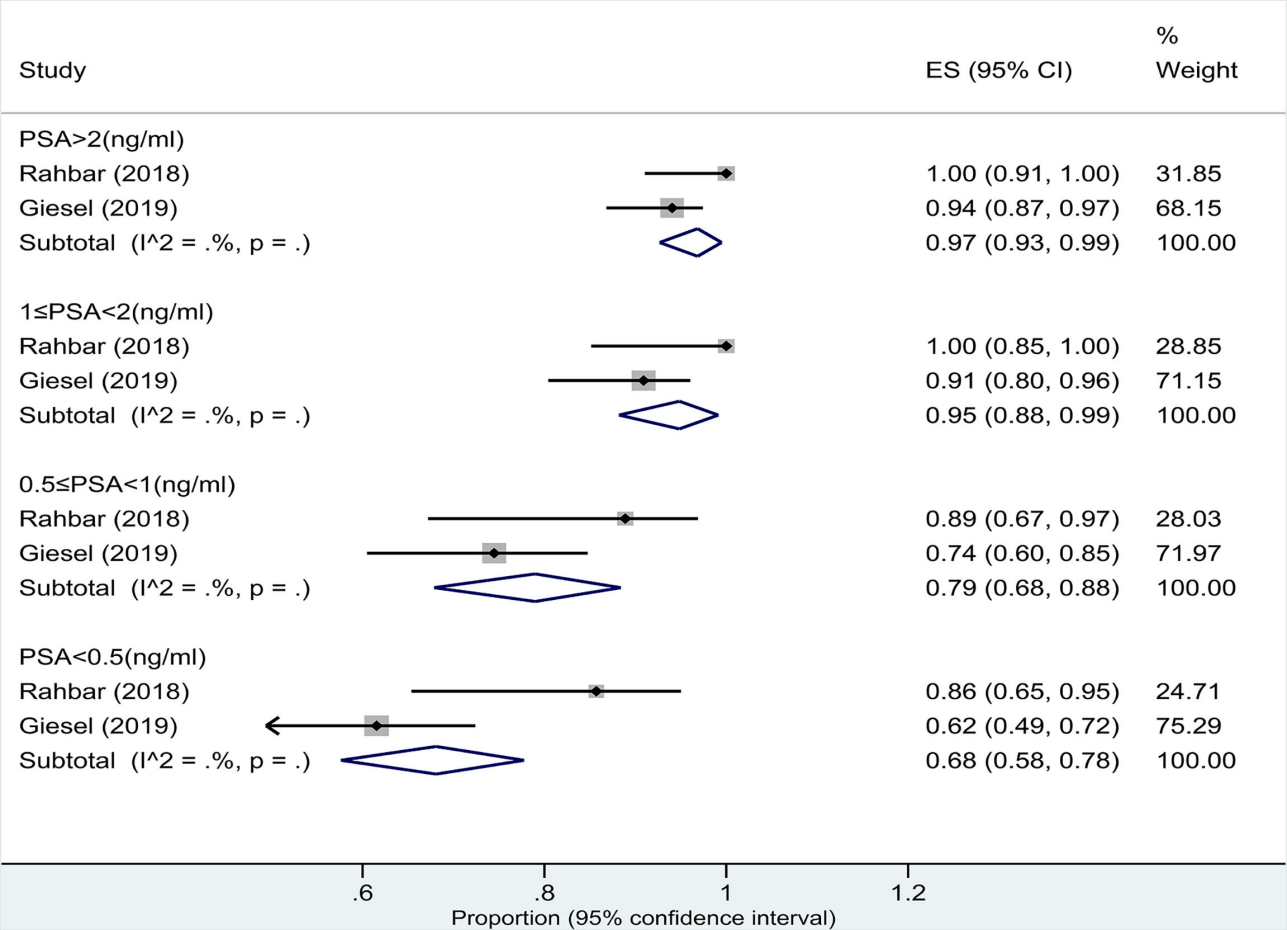
Plot of the pooled detection rate of ^18^F-PSMA-1007 PET/CT for prostate cancer with biochemical recurrence based on patient analysis with PSA levels >2.0, 1.1–2.0, 0.51–1.0, and ≤0.5 ng/ml.

Two studies (15, 16) assessed the pooled DR of ^18^F-PSMA-1007 PET/CT for PCa in BCR based on patient analysis. The DRs of PSA levels >2.0, 1.1–2.0, 0.51–1.0, and ≤0.5 ng/ml detected by ^18^F-PSMA-1007 PET/CT were 97% (95% CI: 93%–99%), 95% (95% CI: 88%–99%), 79% (95% CI: 68%–88%), and 68% (95% CI: 58%–78%), respectively (**Figure 5**). There was a significant difference between the four groups (*P* = 0.00).

##### 3.4.2.3 ^18^F-PSMA-1007 PET/CT vs. 68Ga-PSMA-11 PET/CT

Two studies (11, 37) simultaneously compared ^18^F-PSMA-1007 with ^68^Ga-PSMA-11 PET/CT for PCa in biochemical recurrence. The pooled DRs of ^18^F-PSMA-1007 vs. ^68^Ga-PSMA-11 PET/CT in PCa were 87% (95% CI: 80%–92%) vs. 47% (95% CI: 38%–55%) in a patient-based analysis and 46% (95% CI: 41%–50%) vs. 89% (95% CI: 86%–92%) in a lesion-based analysis, respectively (**Supplementary Figure 4**). The pooled results should be interpreted carefully, given the fact that the results were only based on two studies.

## 4 Discussion

In the previously published meta-analyses (19, 20, 38, 39), Treglia et al. (38) analyzed the DR of ^18^F-labeled PSMA PET/CT for the biochemical recurrence of PCa. In their meta-analysis, four studies were included assessing the application value of 18F-PSMA-1007, with a pooled DR of 89%. However, Treglia et al. (38) did not perform subgroup analyses for each radiotracer at different serum PSA levels. Alberts et al. (40) performed a network meta-analysis on the diagnostic performance of different radiotracers in recurrent prostate cancer and believed that ^18^F-PSMA-1007 had a good advantage in the detection of prostate cancer lesions. However, their study has the following shortcomings: the literature after 2020 was not included, as this was the year when many new studies on ^18^F-PSMA-1007 were published; no grouping of lesions and patients was performed; and the DRs of different serum PSA levels were not analyzed. Therefore, it is necessary to re-conduct a meta-analysis on this background. Our meta-analysis suggested that ^18^F-PSMA-1007 PET/CT has a good DR in patients with different serum PSA levels in the setting of primary staging or BCR of PCa.

In our meta-analysis, the serum PSA was higher than 2 ng/ml in all primary staging patients, and the combined DR of ^18^F-PSMA-1007 PET/CT was 94% (95% CI: 92%–96%). In addition, we performed patient- and lesion-based subgroup analyses, and the pooled DRs of ^18^F-PSMA-1007 PET/CT were 96% (95% CI: 91%–99%) and 81% (95% CI: 66%–92%), respectively. Our study also found that the difference between the two groups was statistically significant (*P* = 0.02). In other words, the DR of the former was significantly higher than that of the latter. Possible reasons for the difference in the results between the two subgroups include the following: first, in a patient-based analysis, a prostate cancer patient who has only one lesion is considered positive. However, in the lesion-based analysis, the number of lesions was large and there were many false-positive lesions, so the true positive rate decreased. Second, the subjects analyzed were not derived from the same study, and not all subjects were head-to-head comparisons. Third, there was significant heterogeneity between the studies in the lesion-based analysis (*I* ^2^: 96.47%). However, in the patient-based analysis, there was no heterogeneity (*I* ^2^: 22.13%). Grünig et al. (41) concluded that ^18^F-PSMA-1007 PET/CT detected specific uptake foci in bone in 51.4% of the patients with prostate cancer. In a recent original study, the overall positive detection rate of ^18^F-PSMA-1007 PET/CT was 91% in the BCR of prostate cancer (42). However, the study also found a significantly lower positive predictive value for ^18^F-PSMA in bone lesions compared to local recurrence and pelvic lymph nodes, which are a potential diagnostic weakness when using this tracer (42). Therefore, it can be concluded that the DR of the lesion-based analysis in this study was lower than that of the patient-based analysis.

Three studies simultaneously compared the DR of ^18^F-PSMA-1007 PET/CT with MRI for PCa in primary staging in a lesion-based analysis. However, our pooled results showed no significant difference in the DR of the two imaging modalities (*P* = 0.409). Kesch et al. (17) believed that ^18^F-PSMA-1007 performed slightly better for near-total agreement regarding sensitivity, specificity, PPV, and accuracy but had a worse sensitivity and NPV for total agreement than the multiparameter MRI (mpMRI). This variance can be explained by the higher resolution and anatomic landmark definition derived from mpMRI. Based on the per-lesion analysis, ^18^F-PSMA-1007 PET/CT was superior to mpMRI, having both fewer false negatives and fewer false positives (17). Our findings are consistent with those of Kesch et al. (17). Furthermore, the study by Privé et al. (30) of 53 patients with primary prostate cancer found ^18^F-PSMA-1007 to accurately stage seminal vesicle invasion (i.e., pT3b) more often than mpMRI (90% vs. 76%), while mpMRI detected extracapsular extension (i.e., pT3a) better than ^18^F-PSMA-1007 (90% vs. 57%).

In this study, the pooled DRs of ^18^F-PSMA-1007 PET/CT in the BCR of prostate cancer were 82% (95% CI: 74%–88%) (per patient) and 78% (95% CI: 33%–100%) (per lesion), respectively. Although the combined DR of the two was not statistically different, the confidence intervals based on the lesion were large, so the reliability of the combined results might be slightly less. In addition, we performed a subgroup analysis of serum PSA in patient-based studies. However, due to the limited amount of data, only two studies (15, 16) were included in the analysis. The pooled DRs of PSA levels >2.0, 1.1–2.0, 0.51–1.0, and ≤0.5 ng/ml detected by ^18^F-PSMA-1007 PET/CT in the BCR of PCa patients were 97%, 95%, 79%, and 68%, respectively. In the meta-analysis of Treglia et al. (38), the authors found the DR of ^18^F-PSMA PET/CT in the BCR of PCa patients with PSA ≥0.5 ng/ml (pooled DR: 86%; 95% CI: 78%–93%) compared to patients with PSA <0.5 ng/ml (pooled DR: 49%; 95% CI: 23%–74%). Therefore, the accurate timing of ^18^F-PSMA PET/CT, based on PSA values, substantially affects its diagnostic value in the BCR of PCa patients, and monitoring of PSA values could be useful for accurate timing of ^18^F-PSMA PET/CT (38). Eiber et al. (43) demonstrated that, as with other PET tracers, the detection rate of PSMA PET/CT increases with the blood level of PSA, showing a detection rate >95% in patients with PSA ≥2 ng/ml. Although only two studies were included in our analysis, the results obtained also showed that ^18^F-PSMA-1007 PET/CT was also better detected in prostate cancer with increased serum PSA levels. This conclusion is consistent with other studies (15, 16, 38, 43).

PSA kinetics has been proposed to supplement other diagnostic modalities in patient selection, especially with low PSA (44). In a 2019 meta-analysis, Pereira Mestre et al. (45) used different PSA doubling times (PSAdt) to assess the DR of PSMA-PET in the biochemical recurrence of prostate cancer. Their results showed that the pooled DR of PSMA-PET in restaging prostate cancer patients was 72%, increasing to 83% when PSAdt was ≤6 months and decreasing to 60% when PSAdt was >6 months. Therefore, they concluded that PSA kinetics, and in particular shorter PSAdt (≤6 months), may be a predictor of PSMA-PET positivity in patients with biochemically recurrent prostate cancer.

There were three studies simultaneously comparing the application of ^18^F-PSMA-1007 and ^68^Ga-PSMA-11 PET/CT in the primary stage (21) and biochemical recurrence (11, 37) of prostate cancer. However, data from only two studies (11, 37) could be included in the meta-analysis. The pooled DRs of ^68^Ga-PSMA-11 PET/CT in PCa were 47% in a patient-based analysis and 89% in a lesion-based analysis, respectively. In a network meta-analysis of the diagnostic performance of radiotracers in recurrent PCa, the results showed a higher DR ^18^F-PSMA-1007 than ^68^Ga-PSMA and ^18^F-DCFPyl with a surface under the cumulative ranking curve of 0.9997 (40). The authors stated their result with caution because only one study (33) was analyzed. Kuten et al. (21) performed a head-to-head comparison of the findings of ^18^F-PSMA-1007 PET/CT and ^68^Ga-PSMA-11 PET/CT in the same patients presenting with newly diagnosed intermediate- or high-risk PCa using histopathology and immunohistochemical staining as reference standards. They showed that both ^18^F-PSMA-1007 and ^68^Ga-PSMA-11 identify all dominant prostatic lesions in patients with intermediate- and high-risk PCa at staging. However, ^18^F-PSMA-1007 may detect additional low-grade lesions of limited clinical relevance. Morawitz et al. (37) compared the PSMA PET/CT and CT alone for the detection of biochemical recurrence of PCa and their effect on treatment. They found that both ^68^Ga- and ^18^F-PSMA PET/CT performed significantly better than CT alone, with almost equivalent *P*-values, suggesting that the diagnostic performances of both tracers are similar. Rauscher et al. (11) showed that the sensitivity of ^18^F-PSMA-1007 PET/CT was significantly higher than that of ^68^Ga-PSMA-11. However, both had the same detection rate for recurrent prostate cancer in patient-based studies. Researchers found that PET/CT with ^18^F-PSMA-1007 detected recurrent lesions more accurately closer to the bladder wall. There was a slightly higher DR for ^18^F-PSMA-1007 at low PSA levels, possibly due to the different energy distributions of ^18^F and ^68^Ga positron emitters (16). Theoretically, the resolution of ^18^F is higher than that of ^68^GA, especially in human PET systems (46). Therefore, it could be hypothesized that ^18^F-labeled PSMA ligands might improve the detection sensitivity for very small tumors (16).

Heterogeneity between studies may represent a potential source of bias in meta-analyses (47). Diversity of patient characteristics, differences in methodology, and overall quality of the study may all be sources of heterogeneity. In our meta-analysis, there was a significant difference between studies (*I* ^2^ > 50%), so the random effects model was used to combine effect sizes. To reduce possible sources of heterogeneity, subgroup analyses were performed according to different serum PSA levels, imaging agents, and imaging devices. Publication bias is a major issue in all meta-analyses, as studies reporting significantly positive results are more likely to be published than studies reporting negative results (48). Indeed, it is not uncommon for small-scale early studies to report a positive relationship that subsequent large studies cannot replicate (47). In our meta-analysis, funnel plot and Egger’s test were used to evaluate publication bias. The funnel plot shows the symmetry of the pooled DR, indicating that there was no publication bias based on the patient and lesion analyses, as confirmed by the results of Egger’s test. In addition, we used the QUADAS-2 tool to evaluate the included studies and found that most were of medium to high quality.

It is important to note that our study has some limitations. First, the DRs of ^18^F-PSMA-1007 and ^68^Ga-PSMA-11 in prostate cancer have only been compared and analyzed in two studies simultaneously, so the combined results need to be interpreted cautiously. Second, in the primary prostate stage group, all included studies had serum PSA >2 ng/ml and did not evaluate the use of ^18^F-PSMA-1007 PET/CT in PSA ≤2 mg/ml. Third, although some of the positive lesions detected by ^18^F-PSMA-1007 PET/CT were considered as biochemical recurrence, those lesions were merely clinically monitored rather than pathologically confirmed. Hence, false positives were not able to be ruled out. Lastly, the study results were heterogeneous. A subgroup analysis was carried out to reduce heterogeneity, but heterogeneity was present across subgroups. This may be related to differences in the study population, methods, quality, and the general lack of appropriate reference criteria. In the future, these shortcomings need to be addressed through large-scale, high-quality, and better-reported studies.

## 5 Conclusion

This meta-analysis concluded that ^18^F-PSMA-1007 PET/CT had a high application value for prostate cancer, including primary tumors and biochemical recurrence. The DR of ^18^F-PSMA-1007 PET/CT was slightly higher in primary prostate tumors than in biochemical recurrence. Our study found that the DR of the ^18^F-PSMA-1007 PET/CT was also improved with increasing serum PSA levels.

## Data availability statement

The original contributions presented in the study are included in the article/**Supplementary material**. Further inquiries can be directed to the corresponding author.

## Author contributions

XL, WBZ, and TJ contributed to the conception and design of the study. XL and YS organized the database. CLG performed the statistical analysis. XL wrote the first draft of the manuscript. XL, TJ, CLG, and HTL wrote sections of the manuscript. All authors contributed to the manuscript revision and have read and approved the submitted version.

## Conflict of interest

The authors declare that the research was conducted in the absence of any commercial or financial relationships that could be construed as a potential conflict of interest.

## Publisher’s note

All claims expressed in this article are solely those of the authors and do not necessarily represent those of their affiliated organizations, or those of the publisher, the editors and the reviewers. Any product that may be evaluated in this article, or claim that may be made by its manufacturer, is not guaranteed or endorsed by the publisher.
