# Supplementary material for: Detection rate of fluorine-18 prostate-specific membrane antigen-1007 PET/CT for prostate cancer in primary staging and biochemical recurrence with different serum PSA levels: A systematic review and meta-analysis
Source: Front Oncol. 2022 Jul 22;12:911146. doi: 10.3389/fonc.2022.911146 (PMC9353183; doi:10.3389/fonc.2022.911146)
Supplement: Supplementary file 1 [file DataSheet_1.docx]

Supplementary Material

## Supplementary Figures


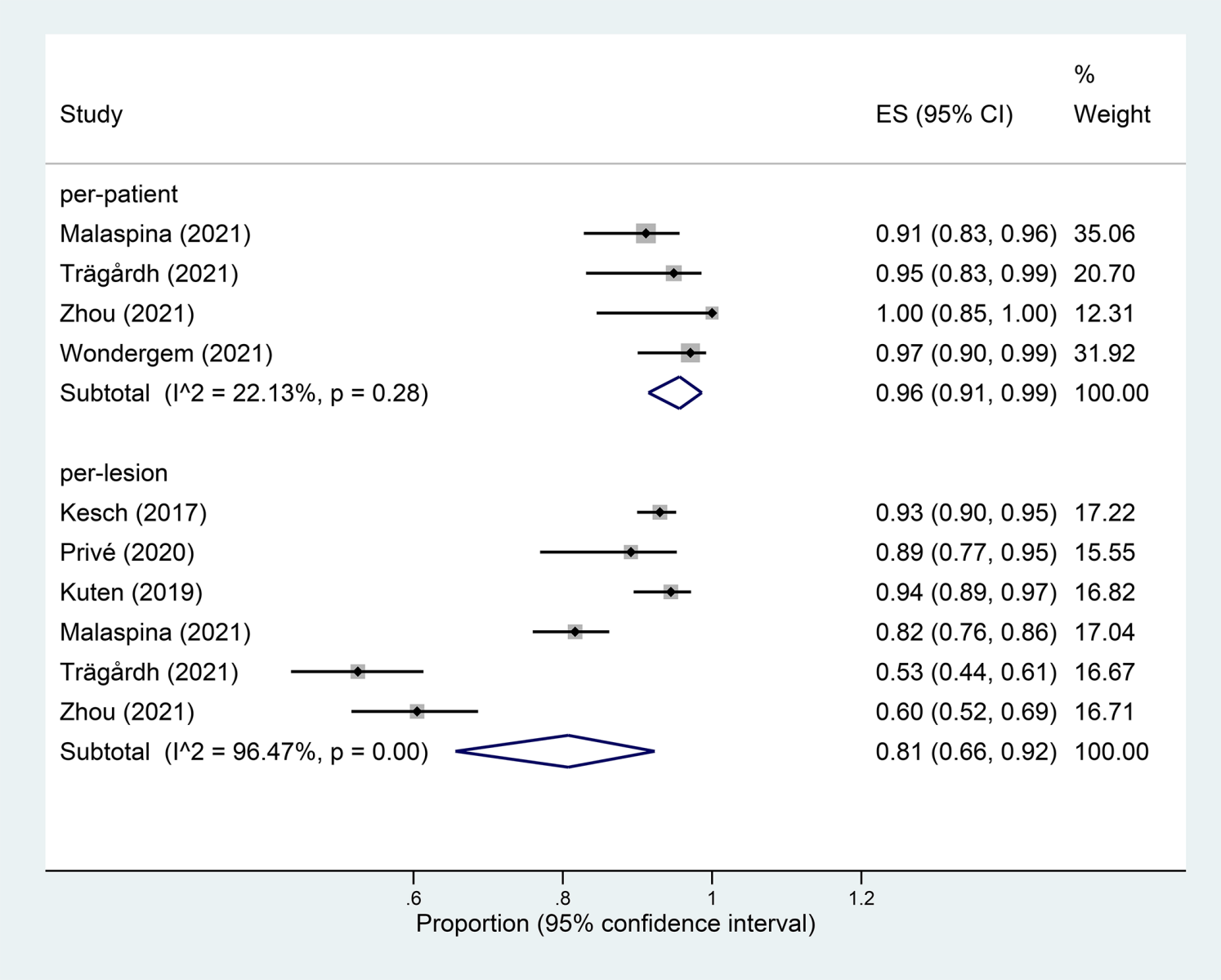


**Supplementary Figure 1.** Plot of pooled detection rate of ^18^F-PSMA-1007 PET/CT for prostate cancer in primary staging based on patient/lesion analysis.

**
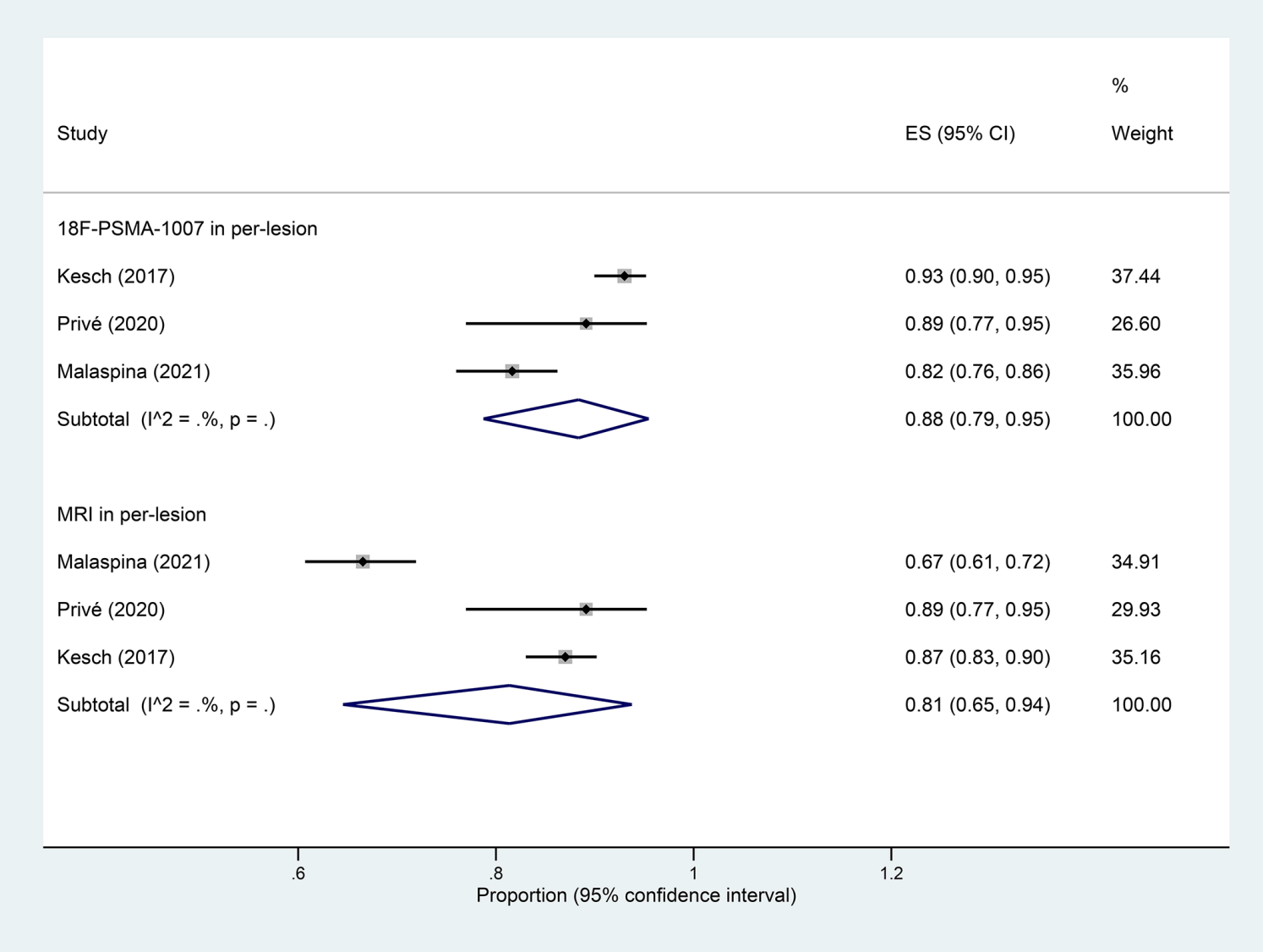
**

**Supplementary Figure 2.** Plot of pooled detection rate of ^18^F-PSMA-1007 PET/CT vs MRI for prostate cancer in primary staging in a lesion-based analysis.


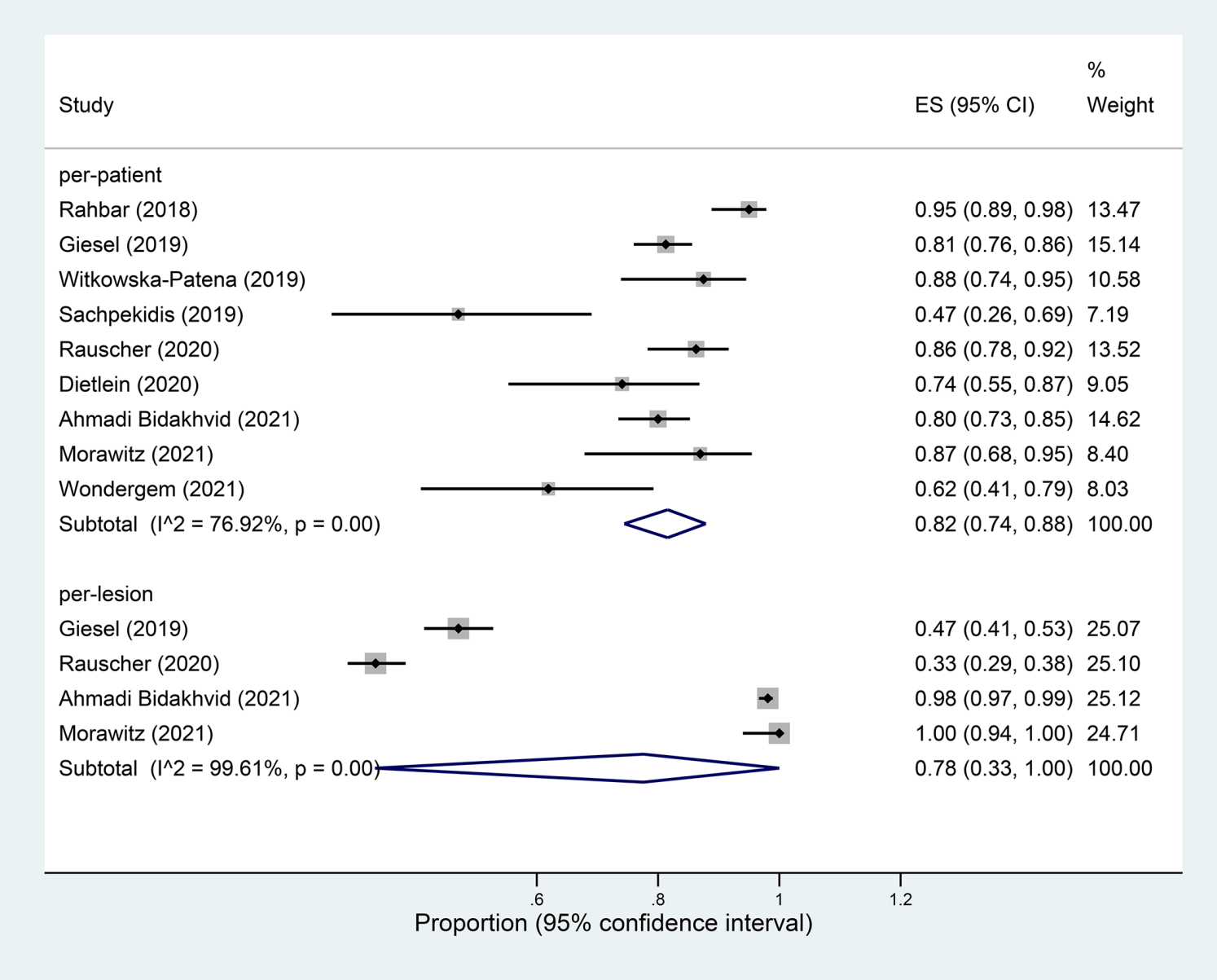


**Supplementary Figure 3.** Plot of pooled detection rate of ^18^F-PSMA-1007 PET/CT for prostate cancer with biochemical recurrence based on patient/lesion analysis without serum PSA grouping.


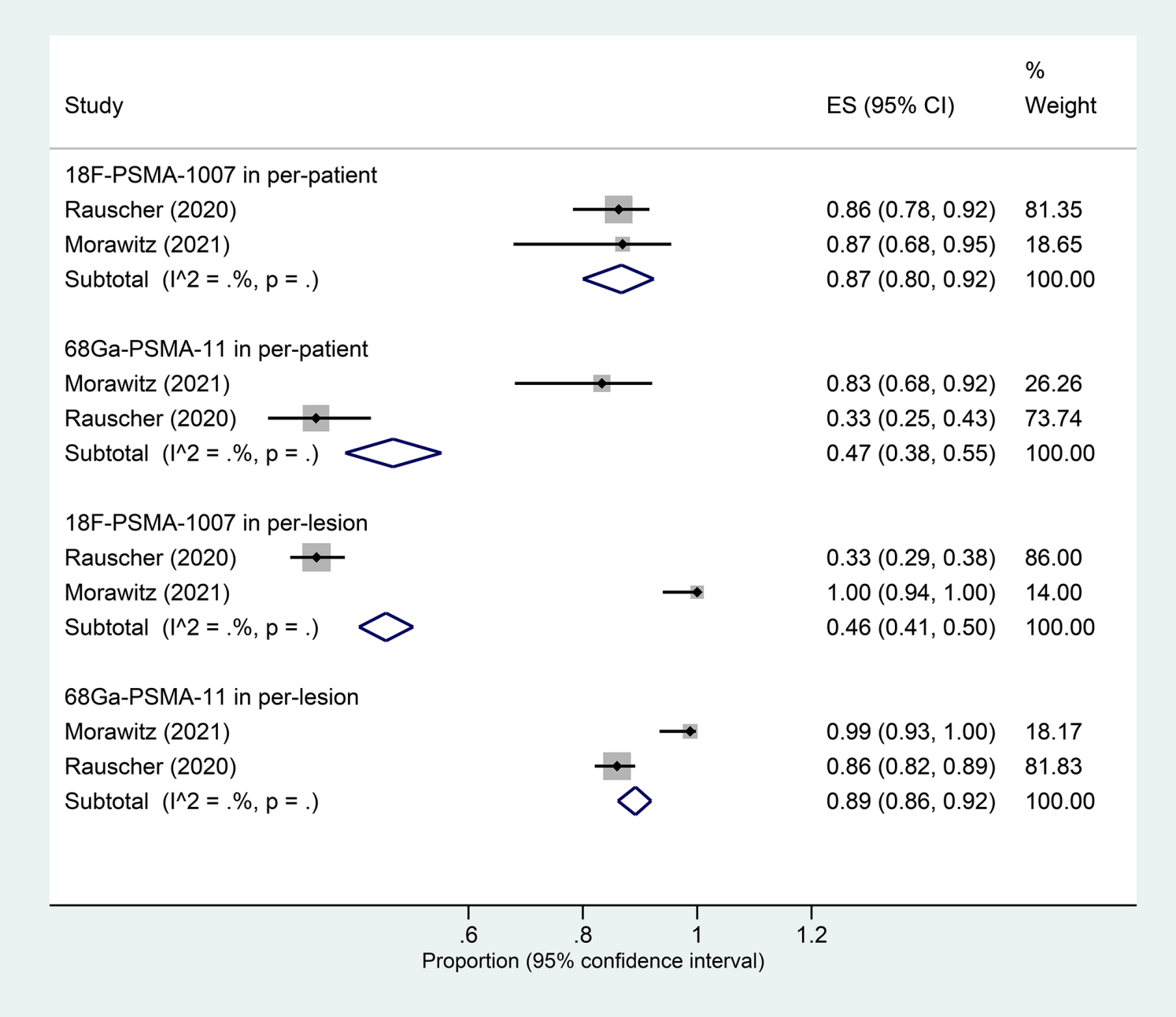


**Supplementary Figure 4.** Plot of pooled detection rate of ^18^F-PSMA-1007 vs ^68^Ga-PSMA-11 PET/CT for prostate cancer with biochemical recurrence based on patient/lesion analysis**.**

**Supplementary Tables**

**Supplementary Table 1.** Review of the quality of the studies included according to the Quality Assessment for Diagnostic Studies-2 (QUADAS-2) tool. Risk of Bias and Applicability Concern for patient selection, index test, reference standard, and flow and timing.

|  | **Risk of Bias** | | | | **Applicability Concern** | | |
| --- | --- | --- | --- | --- | --- | --- | --- |
| **Authors** | **Pt selection** | **Index**  **test** | **Ref**  **Std** | **Flow and**  **timing** | **Pt Selection** | **Index Test** | **Ref Std** |
| Zhou et al.(4) | Low | Low | Low | Low | Low | Low | Low |
| Rauscher et al.(11) | Low | Unclear | Low | Low | Low | Unclear | Low |
| Rahbar et al.(15) | Low | Low | Low | Unclear | Low | Low | Low |
| Kesch et al.(18) | Low | Low | Unclear | Low | Low | Low | Low |
| Trägårdh et al.(19) | Low | Unclear | Low | Low | Low | Unclear | Low |
| Kuten et al.(23) | Low | Unclear | Unclear | Low | Low | Unclear | Low |
| Malaspina et al.(24) | Low | Unclear | Low | Low | Low | Low | Low |
| Privé et al.(33) | Low | Low | Low | Low | Low | Low | Unclear |
| Wondergem et al.(34) | Low | Low | Low | Low | Low | Low | Low |
| Giesel et al.(35) | High | Low | Low | Low | Low | Low | Low |
| Witkowska-Patena et al.(36) | Unclear | Low | Low | Low | Low | Low | Low |
| Sachpekidis et al.(37) | High | Unclear | High | Unclear | Low | Unclear | Low |
| Dietlein et al.(38) | Low | Low | Unclear | Low | Low | Low | Low |
| Ahmadi Bidakhvid et al.(39) | Low | High | High | Low | Low | Low | Low |
| Morawitz et al.(40) | Low | Low | Unclear | Low | Low | Low | Unclear |
